# A Brief Overview of Polymers Science and Technology, in Spain

**DOI:** 10.3390/polym14040652

**Published:** 2022-02-09

**Authors:** Carmen Mijangos

**Affiliations:** Instituto de Ciencia y Tecnología de Polímeros, Consejo Superior de Investigaciones Científicas (ICTP-CSIC), Juan de la Cierva 3, 27006 Madrid, Spain; cmijangos@ictp.csic.es

**Keywords:** polymers in Spain, research groups, Spanish contributions, overview

## Abstract

This Special Issue *State-of-the-Art on Polymer Science and Technology in Spain* is comprised of a collection of 42 publications/contributions related to very different topics undertaken by the numerous research groups working in Spain in Polymer Science and Technology. This monograph collects the contributions of more than 200 different authors from 24 different national Institutions (>30 different centers/departments) from Universities and CSIC centers distributed throughout the whole of Spain. Two-thirds of the contributions to this Special Issue arise from Institutional collaborations, half of which are international collaborations with European research groups and the other half with other international research groups outside Europe including China, Australia or United States of America among others. This brief overview communication provides a general overview of the research lines in Polymer Science and Technology covered in Spain and show most of the representative polymer groups and their distribution throughout Spain. Most of Spanish polymer groups belong to the Grupo Especializado de Polímeros (GEP) being part of the European Polymer Federation (EPF). It also shows how Spanish science about polymers is positioned at European level.

## 1. Introduction

Even if polymer science and technology in Spain started later than in other European countries, nowadays polymers have reached a good position in science and technology rankings all over the world. The creation of two research centers exclusively devoted to polymer research and the development of a high number of polymer research groups in most of the 50 public universities, together with the great number of national and multinational polymer companies established and disseminated throughout Spain, have contributed to important advances in polymer science. On occasion of the 100 years anniversary of Polymer Science in 2020, the journal *Anales de Química of the Spanish Royal Chemistry Society* (RSEQ) has recently published a paper in which the beginnings of polymer science in Spain, which date back to 1947, and the chronological evolution on academia and industry, are documented [1]. Until the 1980s, research groups were funded by their own institutions and/or private companies, research was highly compartmentalized, and researchers practically exclusively worked on very specific topics. This was also the case for other researchers working in different fields other than polymer science [1,2]. It is the period 1980–1990, when new and strong research groups emerged, which resulted in a great expansion of polymers in Spain. In 1985, there was an important milestone: the creation (irruption) of the National Materials Program from the Spanish Ministry of Education and Science and the integration of polymers within the materials research community. As a result, research projects started to be competitive, polymer research groups became more numerous and polymer projects had a relatively high success rate within the division of materials science. Since then, numerous polymer topics have been covered within the research groups, which are distributed throughout Spain, with two important focuses in the Autonomous Community of the Basque Country (CAPV) and the Autonomous Community of Madrid (CAM) and rapid increase in contributions from other autonomous communities, such as, Valencia, Catalonia, Castille-León and Andalusía. During this time, many existing research groups have been consolidated while new and strong polymer groups have emerged.

In the 1990s, another important milestone occurred, the launch of the Europe-wide Framework Programs for research where Spanish laboratories, technological centers and polymer companies began to actively participate and produce relatively important returns. It can be said that, thanks to the emergence of many research groups, polymer science at the national level covers practically the entire spectrum of polymer science and technology. Since the 1990’s, polymer research has been focused on the development of polymers with specific technological properties. In fact, many laboratories are directly involved in the development of polymers for applications through EU and industrial collaborative projects and obtain important returns. For instance, Spain was the second country in the ranking of the NMBP Program of the EU, with 14.4% of the budget [3].

As a consequence, in most of the polymer groups, with a few exceptions, fundamental aspects of polymer science are decreasing in favor of polymer applications. It is important to note that, besides academic groups, there are several specific polymer technological centers connected to industry located throughout Spain.

## 2. Polymers in Spain Today

Most of the research lines related to polymer science and technology are covered by a large number of groups belonging to research institutions and universities, which are distributed throughout Spain. Polymer groups mainly constituted of chemists, engineers, physics and an increasing number of biologists, pharmacists, etc. Nowadays, most of the consolidated polymer research lines deal with (i) *polymer synthesis*: controlled polymerization, polycondensation; polymer functionalization, modelling, polymerization, process engineering, etc.; (ii) *polymer physics*: polymer structure, polymer dynamics, crystallization, gelation, rheology, etc., (iii) *polymers for applications* encompass: biomaterials, food packaging, polymer membranes, transport, sensors, etc. or more recently polymers for a circular economy. Compared to a few years ago, the research lines undertaken by different polymer research groups are continuously evolving, probably mediated by the research projects in which they are involved for which they receive funding. Moreover, there are research lines that are only specific to one or two groups, whereas others are common to many research groups, with the risk of sometimes overlapping one another (as happens all over the world). We can say that Spanish polymer research lines are among the challenges proposed by *Macromolecules* [4] *and Progress in Polymer Science* [5], even if the number of challenges proposed by *Macromolecules* in the opinion of this author is insufficient.

This Special Issue “*State-of-the-Art on Polymer Science and Technology in Spain* represents a detailed overview of recent research developments on fundamental and application-inspired aspects of polymer science in Spain addressed by different polymer research groups. As a whole, it contains 42 contributions, of which 9 are reviews and 33 are articles/communications (including this short overview). Even though this monograph set is not unified by a common approach to identify the most outstanding challenges on polymer science, it is of great help to gathering of all the knowledge behind these works that contribute to the development of novel polymer materials and to understand the most relevant aspects of polymer behavior.

To summarize the content of the Issue, first, it is worth considering the nine reviews, ordered chronologically in term of time of reception. They cover different topics: fundamentals of polymer structure and physics; polymer rheology in Spain over the last 25 years; polyelectrolyte and natural polymer multilayers; polymer self-assembly; conjugated polymers; collagen based-biomaterials and chitosan based-biomaterials; and polymer crystallization. In the following, a short description of each review is given starting with the most general reviews, grouped thematically:

1. (Review 2 [6])—Insight into the Structure and Dynamics of Polymers by Neutron Scattering Combined with Atomistic Molecular Dynamics Simulations, by Arantxa Arbe et al. (CFM-CSIC&UPV/EHU; Basque Country). This review offers a detailed view of the methodology developed by the authors and shows the added value of the combination of neutron scattering and fully atomistic MD-simulations in deciphering the structural properties of chemically simple polymers as well as of other complex systems. The methodology is illustrated with examples covering a wide range of systems. Furthermore, the potential of this strategy is also highlighted in the case of the dynamical studies by showing a substantial increase in the understanding of different processes characteristic of polymers, ranging from the simplest motions (methyl group rotations) to the Rouse dynamics, passing through the local processes involved in the secondary relaxations and the structural relaxation. The authors also address many interesting questions in the field of polymers that remain open and are still challenging, i.e., multi-component systems and/or systems containing macromolecules with complex architectures or displaying relevant features at mesoscopic scales.

2. (R8 [7])—*Rheology of Polymer Processing in Spain (1995–2020*), by Leire Sangroniz et al. (POLYMAT-UPV/EHU and UHuelva; Basque Country and Andalusia). This review is a clear overview of polymer rheology in Spain. The authors report in detail the contribution of Spanish scientists to the rheology involved in polymer processing during the last 25 years. It also provides information related to the first steps in the field of rheology in Spain, the pioneers and the Spanish rheology group (GER, created in 1983). The manuscript is divided into six sections, corresponding to different families of industrial polymers: thermoplastics, thermosets, adhesives, biopolymers, composites and nanocomposites, and polymer-modified bitumen. Moreover, the rheological behavior of these materials in processing methods such as extrusion, injection molding, additive manufacturing, and others is discussed, based on the literature results. A detailed qualitative and quantitative view of the most outstanding achievements, based on the rheological criteria of the authors, is also offered.

Other contributions related to rheology of polymer gels, a subject of initial collaboration between POLYMAT (UPV/EHU) and ICTP-CSIC (Antxon Santamaria and Carmen Mijangos) are not included in this section [8,9], probably because they will be reported in the Special Issue of *Polymers*, entitled *Rheology Applied to Polymer Characterization and Processing. A Themed Honorary Issue to Prof. Antxon Santamaria* [10].

3 and 4. Two reviews focused on the layer by layer methodology (R5 [11] and R6 [12]). Polyelectrolyte Multilayers on Soft Colloidal Nanosurfaces: A New Life for the Layer-By-Layer Method by Ana Mateos-Maroto et al. (UCM; Madrid) and Multilayer Films from Natural Polymers as Platforms for Biomedical Applications by Miryam Criado-Gonzalez et al. (ICTP-CSIC; Madrid). Both reviews are complementary. The first discusses some fundamental aspects related to deposition methodologies commonly used for fabricating LbL materials on colloidal templates together with the most fundamental physicochemical aspects involved in the assembly of LbL materials. Furthermore, it proposes an analysis of some of the current trends on the fabrication of LbL materials using soft colloidal nanosurfaces, including liposomes, among others. The second review collects the main advances concerning multilayer assembly of natural polymers employing the most used LbL techniques, i.e., dipping, spray and spin coating, leading to multilayer polymer structures and the influence of several variables, i.e., pH, molar mass, and method of preparation, in this LbL assembly process. Finally, the review reports the employment of these multilayer biopolymer films in selected biomedical applications (i.e., platforms for tissue engineering, drug delivery or thermal therapies).

5. (R1 [13]) *Directed Self-Assembly of Block Copolymers for the Fabrication of Functional Devices* by Christian Pinto-Gómez et al. (IMM- CSIC; Catalonia). This paper reviews the main principles of directed self-assembly of block copolymers and gives a brief overview of some of the most extended applications. In particular, the method as a patterning option for the fabrication of nanoelectromechanical systems and the corresponding proof of concept are considered.

6. (R3 [14])—*Aggregation-Induced Emission Properties in Fully-Conjugated Polymers, Dendrimers, and Oligomers* by Antonio Sánchez-Ruiz et al. (UCLM; Castille La Mancha) This review is focused on the synthesis of selected fully conjugated oligomers, dendrimers and polymers in which luminogen units are repeated and combined to yield compounds with aggregation-induced emission or aggregation induced emission enhancement properties. It briefly summarizes the synthetic routes, fluorescence properties and potential applications. Furthermore, an exhaustive comparison between spectroscopic results in solution and aggregates or in the solid state is collected in almost all examples, and an opinion on the future direction of the field is briefly stated by the authors.

7. (R4 [15])—*Collagen Type I Biomaterials as Scaffolds for Bone Tissue Engineering* by Gustavo A. Rico-Llanos et al. (U Malaga and Universidad Nacional de Colombia; Andalusia y Colombia). This review summarizes the current status of collagen type I as a biomaterial for bone tissue engineering and highlights some of the main efforts that have been recently made towards the design and production of collagen implants to improve bone regeneration. The review also includes a list of different forms of collagen-based biomaterials for several tissue engineering applications. Furthermore, it also addresses the future research in this field.

8. (R9 [16])—*Chitosan: An Overview of Its Properties and Applications,* by Inmaculada Aranaz et al. (UCM, Madrid). In this review, the authors discuss how chitosan chemistry can solve some of the problems related to its poor solubility and boost the polymer properties and applications. In particular, some of the main biological properties of chitosan and the relationship with the physicochemical properties of the polymer are considered. In addition, the authors review the use of chitosan in the green synthesis of metallic nanoparticles and its use as support for biocatalysts and finally, they briefly describe the use of chitosan-based systems for drug delivery.

9. (R7 [17])—*Synthesis, Structure, Crystallization and Mechanical Properties of Isodimorphic PBS-ran-PCL Copolyesters***,** by Maryam Safari et al. (POLYMAT-UPV/EHU; Basque Country). This article is focused on the synthesis, structure, crystallization behavior and mechanical properties of isodimorphic random biodegradable copolyesters based on poly (butylensuccinate) and poly (caprolactone). It also provides a comprehensive analysis of the main recent results on PBS-ran-PCL random copolyesters with different molecular weights. This work also shows that the comonomer composition and crystallization conditions are the major factors responsible for the crystalline morphology, crystallization kinetics and mechanical performance of isodimorphic random copolyesters. In this work, the relationships between the comonomer composition and mechanical properties are also addressed 9. (R9 [16]).

Secondly, the themes of the other 32 articles of this Issue reporting the recent advances in several fields of polymer science and technology (this overview not included) are briefly outlined in Table 1. Since contributions are chronologically placed, under author criteria, articles are compiled in four general research topics. (i) *Polymer Synthesis/Fabrication:* synthesis/preparation of new monomers, polymers and composites, with 16 contributions; (ii) *Polymers for applications:* biopolymers, sustainable polymers and bio-applications, with eight contributions; (iii) *Specific preparative routes,* with five contributions and (iv) *Fundamental aspects of polymers science,* with two contributions. In order not to enlarge the table, only a few representative words have been selected from the title or abstracts. The table includes the name of the first author and the name of corresponding or representative author (reviews are not included).

As a whole, this monograph collects the contributions of nearly 200 different authors that belong to 24 different national Institutions (>30 different centers/departments) from Universities and CSIC distributed throughout the whole of Spain. It is important to remark than two-thirds of the contributions arise from collaborations between two or more Institutions, half of which involve European research groups and the other half involving international collaborations with research groups outside the EU, or from China, Australia or the USA.

Even though many of the consolidated research groups in Polymer Science in Spain have contributed to this Special Issue, it only gives partial information of all the polymer themes that are currently being investigated by Spanish teams and, therefore, it does not represent the broad polymer knowledge acquired along many years of research in Spain. In fact, some of the manuscripts published by well consolidated polymer groups report on very specific parts of their research and not the whole activity of the group. In addition, there are many contributions from research groups that are not exclusively working on polymers or have not been working in the field for a long period of time, but are producing outstanding results in the wide spectrum of polymer science

**Figure 1:**
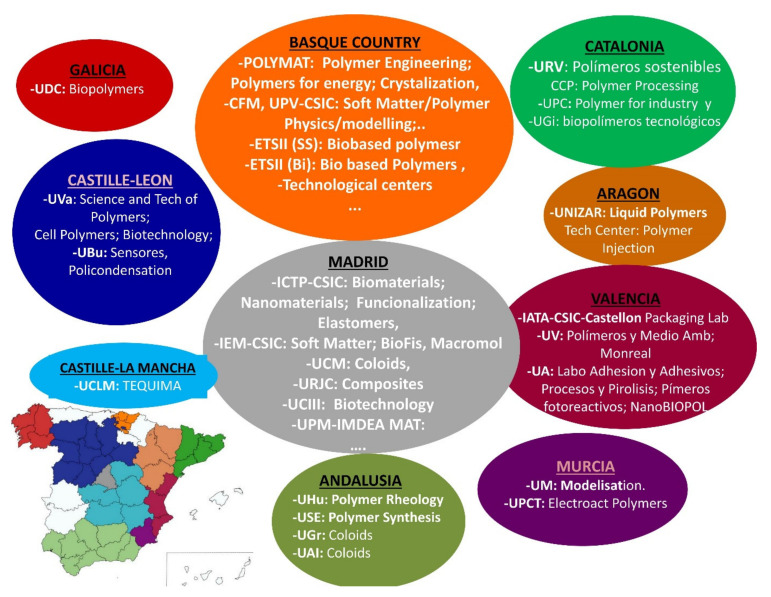
Geographical distribution of polymer groups throughout the local communities of Spain. Reproduced with permission of Anales de Química de la RSEQ [1]. Each color is associated to a local community; see the map of Spain in the left-bottom part for the geographical location.

Nevertheless, the considerable effort made by the sum of individual teams has contributed to promotion of polymers R+D in Spain to a European competitive level. It is important to note that, nowadays, polymer research groups in Spain are distributed all around the autonomous/local communities, see Figure 1. For more detailed information, see [1].

Most polymer researchers are also involved in the training and formation of PhD, MSc and BSc students and experts (every year, ~50 students receive their PhD in polymer science in Spain), which constitutes highly specialized personnel with a lot of demand from private companies and research institutions all over the world. It is worth mentioning some specific courses and two official Master’s Degrees specific to polymer science that are currently being taught in POLYMAT and ICTP-CSIC, those being the High Specialization Master’s degree in Plastics and Rubber (ICTP-CSIC) the first specialized course on Polymer Science in Spain (starting in 1959).

It is important to add that, although this overview is focused on the R+D polymer research from universities and scientific institutions, polymer science and technology in Spain has a network of specific Polymer Technology Centers (or centers which are mostly dedicated to them) attached to different communities and distributed throughout the geography (i.e., AIMPLAST; AIITIP; CIDAUT, CIDETEC, GAIKER and others).

Related to technology of polymers in Spain, it is worth showing some figures. The plastics production and transformation industry in our country accounts for almost 50% of the chemical industry. There are 2200 plastics transformation companies with more than 100,000 workers and 350 plastic raw material manufacturer companies with 11,000 workers [50]. This large production of polymers in Spain is due, on the one hand, to the establishment of many of the large multinational companies within the country and, on the other hand, to the fact that Spain is the first/second largest automobile manufacturer in Europe, an industry that is a large polymer consumer.

## 3. Polymers in Spain within the European, Latin American and International Communities

Within this general polymer science framework, it is worth highlighting the creation of scientific associations related to Polymers in Spain (similar to the ones existing in other countries) and the active participation of Spanish laboratories in the association activities. El Grupo Especializado de Polímeros—The specialized group of polymers (GEP), see the webpage in [51], emerged in 1986 within the Spanish Royal Society of Chemistry (RSEQ) and Spanish Royal Society of Physics (RSEF). Since then, it has promoted the collaboration between different polymer research groups through the organization of different events. As a result of the active participation of a large number of members in various activities and a governing board dedicated to maintaining very active initiatives around the specialized group of polymers, GEP is the second largest group in terms of number of members and one of the most active groups in the RSEQ.

Every two years, chaired by a Spanish polymer team, GEP organizes four-day meetings for all the scientists of the polymer community with the participation of industrial partners in different towns of the country. The GEP meetings have been held uninterruptedly since 1989, except for the case of GEP-SLAP2020 that had to be postponed to 2022 due to COVID-19. The next meeting, GEP-SLAP 2022 (see webpage in [52]), refers to the joint Spanish–Latin Americano meeting, i.e., the XVI Conference of the Grupo Especializado de Polímeros (**GEP2022**) and the XVII Simposio Latinoamericano de Polímeros (**SLAP 2022**)

GEP also promotes the meetings of young polymer researchers (JIP), beginning in 2002 with the 1st National Congress of young researchers in La Manga (Murcia) and, since then, on a biennial basis. It is worth highlighting the joint Spanish (JIP) and French (JEPO) young polymer research congress in 2015, the JIP/JEPO 2015 Conference, in San Sebastián. It also maintains an important recognition for the best theses produced annually by young members of the GEP. GEP community also joints the biennial Symposium Latinoamericano de Polímeros (SLAP).

GEP participation in international actions organized in the framework of the European Polymer Federation, EPF, in which actively collaborates in the organization of European Polymer Congress, every two years, as well as in the specialized schools that are organized is noteworthy. For instance, in 2011, Granada University and ICTP-CSIC hosted the 13th EPF Congress, bringing together more than 1300 european and worldwide polymer researchers.

Spain has also promoted the associations of the Plastics industry: the Spanish Plastics Center (CEP) since 1953, Barcelona; National Consortium of Rubber Industries (COFACO) since 1948 and the Spanish Association of Plastic Industries (ANAIP) since 1957, both in Madrid. Recently (2009), the Spanish Federation of Industries for the Transformation of Rubber and Plastics (FEITCAP) has been created, made up of the two previous associations. National associations are also members of the European trade associations with centers in Brussels, Frankfurt, London, Madrid, Milan and Paris. Spain is also an active member of Plastics Europe, the Association of Plastic Manufacturers.

Within this general context of polymer research in Spain, the continuous growth of polymer groups has aided to the Spanish research reach a place between the first European countries, at least in terms of publications. Spain is placed in the fourth position after Germany, the United Kingdom and France. From 1996 to 2020 Spain has published 12,024 documents. These figures are collected from the SCIMAGO Journal and country rank database [53], searching by the following categories: Material Science; Polymers and Plastics; Western Europe; 1996–2020. There are no data prior to 1996. It is worth noting that, in our country, every 10 years, the number of publications on polymers has doubled and there has been a constant increase in the quality of publications.

## 4. Conclusions

In Spain, polymer research is growing and polymer groups are extending throughout the whole geography. There are two important and specific research polymer centers and many consolidated and strong groups in practically all public and some private universities. Besides polymer research groups there are several technological centers, large polymer manufacturing companies and many small/medim manufacturing and processing companies

Based on established (taking into account) scientific and economical rankings, the polymer scientific level is equal to or higher than the economical level of the country in the EU ranking.

## Figures and Tables

**Table 1:** List of 32 articles, collected by the name of the first author, the name of the corresponding author (and other representative authors) and some representative words.

First Author	Corresponding Author	Representative Words	
		***Synthesis of new monomers, polymers and composites***	
Marta Guembe-García	José Miguel García, Saúl Vallejos	New monomers derived from methacrylamide for amino acid polymer sensors	[18]
Esther Udabe	Maria Forsyth, David Mecerreyes	Ionic liquid monomers covalently integrated into an acrylic polymer coating for corrosion inhibition	[19]
Jorge González-Rodríguez	Jaime González-Álvarez, Belén Altava, Santiago, Luis	Silica-supported polymeric ionic liquid from a vinylic L-valine monomer and divinylbenzene as the chromatographic stationary phase	[20]
Javier Quílez-Bermejo	Diego Cazorla-Amorós	Synthesis of doped polyaniline for mesoporous carbon material	[21]
Beatriz Martínez-Sánchez	Emilia Morallón	Syntheis of phosphonated ring-substituted PANIs: electrochemical properties and computational calculations	[22]
Estefania Fernandez-Bartolome	Rodrigo González-Prieto, Reyes Jiménez-Aparicio	Coordination polymers from heteronuclear dirhodium–gold anionic complexes. Synthesis and crystal structure	[23]
Alexandra Muñoz-Bonilla	Marta Fernández-García	Hidrogels from new 2-hydroxyethyl methacrylate and a methacrylic monomer containing a lateral thiazole group with antimicrobial behavior	[24]
Francesco Gamardella	Angels Serra	Organocatalyzed poly(thiourethane) covalent adaptable networks able to be reshaped and recycled	[25]
Francesco Gamardella	Silvia De la Flor	New family of shape-memory actuators based on poly(thiourethane) networks: thermomechanical properties and shape-memory ability	[26]
Cenit Soto	Pedro Prádanos, Antonio Hernández	Porous organic polymer inclusions within o-hydroxypolyamides containing m-terphenyl moieties for gas separation	[27]
Alberto Concellón	José Luis Serrano	Dendritic macromolecules containing fluorescent coumarin moieties as drug delivery nanocarrier	[28]
Felipe de la Cruz-Martínez	José A. Castro-Osma, Agustín Lara-Sánchez	Ring-opening copolymerization of cyclohexene oxide and zinc catalyzed cyclic anhydrides. Optimization of the process	[29]
Maria Fernández-	Francisco Velasco, Emanuel M. Fernandes	Polyamide powders functionalized with nanosilica for wear resistence applications	[30]
Ángel Alvaredo-Atienza	Juan P. Fernández-Blázquez	Fabrication and characterization of PEEK/PEI multilayer composites by alternate layer stacking	[31]
David Loaeza	Orlando Santana Pérez, Maria Lluïsa Maspoch	Polymeric blends prepared from two recycled plastics, polypropylene and opaque poly(ethylene terephthalate), acting as reinforcement	[32]
Vanessa García	Vanessa García-Martínez, Alejandro Ureña	Aerospace grade benzoxazine resin by means of grapheme nanoplatelet addition	[33]
		***Biopolymers, sustainable polymers and bio-applications***	
Mireia Andonegi	Koro de la Caba, P Guerrero	A green approach towards the preparation of native collagen scaffolds and environmental and physicochemical assessment	[34]
María Blanco	María Blanco	Collagens from European hake and Blue shark skin: differences in the subunit composition influencing the crosslinking pattern.	[35]
Angela Varela-Garcia	Carmen Alvarez-Lorenzo	Design of hydrogel contact lenses endowed with an affinity for acyclovir and its prodrug valacyclovir	[36]
Jone Uranga	Pedro Guerrero, Koro Caba	exopolyssacharides derived from vegetal sources incorporated into protein isolated with antifungal/fungistatic activity	[37]
Francesc X Espinach	Francesc X Espinach	Bio-polyethylene matrix reinforced with fibers from orange tree prunings with notable tensile propertie	[38]
Antonio M. Borrero-López	Concepción Valencia	Cellulose pulp- and castor oil-based polyurethanes for lubricating applications	[39]
Ana M. López-Fernández	Rosa de Llanos, Francisco Galindo	Photoactive materials generating O_2_ by non-covalent rose bengal encapsulation in PHEMA hidrogel films	[40]
Manuel Toledano-Osorio	Manuel Toledano	Doxycycline-doped polymeric membranes to induce growth, differentiation and expression of antigenic markers of osteoblasts	[41]
Gerardo Asensio	B Vazquez, M R Aguilar, L Rojo	Biomimetic gradient scaffolds containing hyaluronic acid and Sr/Zn folates for osteochondral tissue engineering	[42]
		***Specific preparative routes***	
Miriam Abad	Luis Oriol, Víctor Sebastián	Fabrication of supramolecular functionalizable linear–dendritic block copolymer nanocarriers by microfluidics	[43]
Enric Casanova-Batlle	Joaquim Ciurana	Direct ink writing to create printable materials. A revision of the cardiovascular medical application	[44]
María Núñez	Sebastián Muñoz-Guerra and Antxon Martínez de Ilarduya	Enzymatic synthesis of poly(butylene succinate-co-caprolactone) copolyesters	[45]
Irene Cárdaba	Irene Cárdaba, David Mecerreyes	UV-photopolymerization to easily make polymer hydrogels with cleaning properties	[46]
Alba Martínez	Carlos Sánchez-Somolinos	Photolithographic technique to prepare surface relief polymer structures	[47]
		***Fundamental aspects of polymers science***	
A. García-Collado	A. García-Collado	Application of the finite element method to the incremental formation of polymer sheets: the thermomechanical coupled model	[48]
Marta Romay	Nazely Diban	Thermodynamic modeling and validation of the temperature influence in ternary phase polymer systems	[49]

## Data Availability

Not applicable.
